# Prevalence of prolonged grief disorder and its symptoms among bereaved individuals in China: a systematic review and meta-analysis

**DOI:** 10.1136/gpsych-2023-101216

**Published:** 2024-03-05

**Authors:** Meng-Di Yuan, Jun-Fa Liu, Bao-Liang Zhong

**Affiliations:** 1 Research Center for Psychological and Health Sciences, China University of Geosciences (Wuhan), Wuhan, Hubei, China; 2 Department of Psychiatry, Wuhan Mental Health Centre, Wuhan, Hubei, China

**Keywords:** Prevalence, Trauma and Stressor Related Disorders, Psychiatry, Meta-Analysis as Topic, Mental Health

## Abstract

**Background:**

The prevalence of prolonged grief disorder (PGD) and its symptoms among the bereaved population in China vary considerably.

**Aims:**

This meta-analysis aims to estimate the prevalence of PGD and its symptoms among bereaved individuals in China.

**Methods:**

We conducted a literature search in major Chinese and English databases from their inception to 4 October 2023, for cross-sectional studies on the prevalence of PGD or its symptoms in bereaved Chinese individuals. The risk of bias of the included studies and certainty of the evidence were assessed using the Joanna Briggs Institute Critical Appraisal Checklist for Studies Reporting Prevalence Data (‘JBI checklist’) and the Grading of Recommendations, Assessment, Development and Evaluations (GRADE), respectively. The ‘metaprop’ package in R V.4.1.2 was used to synthesise the prevalence.

**Results:**

A total of 28 studies involving 10 994 bereaved individuals were included in the analysis, with JBI checklist scores between 3 and 7. The combined prevalence (95% confidence interval) of PGD and its symptoms was 8.9% (4.2% to 17.6%) and 32.4% (18.2% to 50.8%), respectively. PGD and its symptoms were most prevalent among those who had lost their only child (22.7%) and those bereaved by earthquakes (80.4%), respectively. The GRADE system assigned a very low certainty level to the evidence for the pooled prevalence of PGD and its symptoms.

**Conclusions:**

The pooled prevalence of PGD and its symptoms indicate a potential high need for grief counselling services among bereaved individuals in China. This need is particularly pronounced in those who have lost their only child and those bereaved due to earthquakes. Further methodologically rigorous studies are needed to provide more accurate prevalence estimates.

**PROSPERO registration number:**

CRD42023432553.

**WHAT IS ALREADY KNOWN ON THIS TOPIC**

- China currently has the second-largest annual number of fatalities globally, leading to a substantial number of individuals experiencing the loss of their loved ones. However, reported prevalence rates for prolonged grief disorder (PGD) and its symptoms among bereaved individuals in China vary considerably, ranging from 0% to 37.8% and 2.5% to 88.9%, respectively.

**WHAT THIS STUDY ADDS**

- This meta-analysis found that the pooled prevalence rates of PGD and its symptoms among bereaved individuals in China were 8.9% and 32.4%, respectively. Both rates were lowest among those who lost loved ones to illnesses other than AIDS and COVID-19, while higher rates were observed among individuals who experienced the loss of their only child or a loved one due to earthquakes, AIDS or COVID-19.

**HOW THIS STUDY MIGHT AFFECT RESEARCH, PRACTICE OR POLICY**

- Given the inadequate provision of grief counselling services, the large number of bereaved individuals and the high prevalence of PGD and its symptoms among the bereaved, there is a pronounced unmet need for grief counselling services for the bereaved population in China. Potential public health strategies to address this issue include regular screening for PGD among the bereaved, integrating grief counselling and social work services into primary care settings, establishing a two-way referral system between higher-level mental health institutions and primary care clinics and providing training to primary care and social workers to manage PGD. It is crucial to prioritise the allocation of these service resources to individuals who have lost their only child or experienced the loss of a loved one due to earthquakes, AIDS or COVID-19.

## Introduction

China, the world’s most populous country for several decades, had the highest number of fatalities globally until being surpassed by India in 2021–2022. From 2018 to 2022, the annual number of deaths in China ranged from 9.9 to 10.5 million.^1^ Bereavement, caused by the death of a significant other, is one of the most common and distressing life events. While it is difficult to provide an exact number of individuals experiencing grief after losing a loved one, the number of those bereaved in China could potentially exceed several times the number of deaths. For instance, a single suicide death may leave up to 135 people mourning, while a COVID-19-related death may result in approximately nine family members experiencing bereavement.^2 3^

Bereavement has been linked to an increased risk of physical health issues, emotional and cognitive problems, behavioural issues and impaired social functioning.^4^ While most individuals adapt to the loss over time, a small subset of bereaved individuals experience persistent and intense grief, which may develop into prolonged grief disorder (PGD). PGD is distinguished by persistent and intense yearnings for the deceased, with distinct clinical features different from those seen in major depression and post-traumatic stress disorder.^5^ Although PGD has been provisionally included in the Diagnostic and Statistical Manual of Mental Disorders, Fifth Edition (DSM-5) and formally added to the International Classification of Diseases and Related Health Problems, 11th Edition (ICD-11), controversies persist regarding its clinical symptomatology and diagnostic criteria.^6^ Specifically, the cross-cultural adaptation of the two diagnostic criteria to Chinese societies remains inadequate.^7 8^

Culture plays a significant role in shaping various aspects of mental health, including the sources of distress, the experience of mental disorders, symptomatology, the interpretation of psychiatric symptoms, help-seeking behaviours for mental health problems and the social response to distress.^9 10^ In Chinese culture, bereaved individuals tend to deny the reality of death and suppress their grief, particularly if the deceased is not an older adult.^11 12^ Chinese individuals who have lost a spouse or child may be unfairly stigmatised as unlucky and even blamed for the death, leading to an increased level of social isolation and stigma.^13 14^ This cultural context may increase the risk of PGD and create substantial barriers to accessing grief counselling services for bereaved individuals in China.

In recent years, there has been an increasing research interest in the epidemiology and clinical characteristics of PGD in bereaved individuals in China. However, reported prevalence rates (PRs) for PGD and its symptoms have shown a wide variation, ranging from 0% to 37.8% and 2.5% to 88.9%, respectively.^15–18^ Additionally, studies on high-risk subgroups yielded inconsistent findings. For example, a study conducted by Wang found a similar prevalence of PGD in bereaved men and women (8.5% vs 10.3%, p=0.454), whereas Yi and colleagues reported a significantly lower prevalence of PGD in bereaved men than women (6.6% vs 9.8%, p=0.033).^19 20^ The inconsistent findings on the prevalence of PGD and its high-risk subgroups may be related to the small sample sizes in prior studies, necessitating a meta-analysis for further insights.

In China, two prior meta-analyses have investigated the prevalence of grief in bereaved individuals.^21 22^ However, both studies have certain limitations. One focused only on both PGD and its symptoms in Chinese parents who lost their only child,^21^ while the other solely investigated PGD symptoms in bereaved individuals.^22^ Furthermore, the latter meta-analysis combined prevalence data for PGD and its symptoms without considering their clinical heterogeneity and did not identify high-risk subgroups.^22^ In general, individuals who experience grief symptoms that persist and become clinically significant are more likely to develop PGD. Therefore, it is important to focus on PGD symptoms as they have potential implications for the early prevention of PGD. Studies that examine both PGD and its symptoms are likely to yield more interesting findings. Therefore, we conducted a meta-analysis that considers various causes of bereavement, distinguishes between PRs of PGD and its symptoms and examines high-risk subgroups among bereaved individuals in China.

## Methods

We reported this systematic review and meta-analysis in compliance with the Preferred Reporting Items for Systematic Reviews and Meta-Analyses guidelines (online supplemental material appendix 1),^23^ and the protocol was registered in the International Prospective Register of Systematic Reviews. The literature search, study selection, data extraction, risk of bias (RoB) assessment and certainty assessment of the available evidence were conducted independently by the first and second authors. Disagreements were resolved through mutual agreement.^24^

10.1136/gpsych-2023-101216.supp1 Supplementary data

### Literature retrieval

We conducted a literature search across major Chinese and English bibliographic databases from their inception until 4 October 2023. The databases searched included China National Knowledge Infrastructure, Wanfang Data, SinoMed, VIP Information, PubMed, Embase and PsycInfo. The search terms used were ‘(grief*) AND (bereave* OR mourn* OR sorrow OR surviv* OR Shidu OR los* OR death OR die* OR suicid*) AND (Chin* OR Taiwan OR Hong Kong OR Macau)’. Additionally, we manually searched the reference lists of related literature to ensure that no potential studies were overlooked. Details of the literature search strategies are provided in online supplemental material appendix 2.

### Inclusion and exclusion criteria

The inclusion criteria for the present study were as follows: (a) cross-sectional surveys or baseline data of cohort and interventional studies that reported the prevalence of PGD and/or its symptoms; (b) participants must be Chinese individuals who have experienced bereavement due to various reasons, including suicide, earthquake and illness; (c) standardised tools, such as the 13-item Prolonged Grief Disorder and the Inventory of Complicated Grief, should be used to assess the presence of PGD and its symptoms. PGD is a mental disorder characterised by an intense and prolonged grieving process that significantly impairs functioning and well-being. Diagnosis can be made using existing diagnostic criteria like ICD-11 and DSM-5 or criteria proposed by researchers, such as the Prigerson *et al*’s criteria and the Maciejewski *et al*’s criteria. Symptoms of PGD typically include intense yearning and longing for the deceased, emotional distress, difficulty accepting the death and an inability to move forward in life. These symptoms should be assessed using validated scales, such as the Inventory of Complicated Grief. If multiple studies using the same data were available, only the study with the most complete data was included. Conference papers, reviews and case reports were excluded as well as studies focusing on anticipatory grief or bereavement, resulting from the ending of a significant relationship, abortion, stillbirth or the loss of a pet.

### Data extraction

Information extraction was performed using a predesigned spreadsheet, which captured the following data: (a) basic study details, such as first author, year of publication, study site and survey date; (b) study characteristics, including sampling method, sample size, mean age of participants and outcome assessment; (c) data related to the bereaved individuals, such as PRs or symptom severity scores based on sex and marital status; (d) data related to the deceased individuals, such as PRs or symptom severity scores based on the cause of death; (e) outcome data such as PRs of PGD and its symptoms.

### RoB assessment of included studies

The RoB of the included studies was assessed using the Joanna Briggs Institute (JBI) Critical Appraisal Checklist for Studies Reporting Prevalence Data (hereafter abbreviated as the ‘JBI checklist’).^25^ Nine items covering four domains of RoB in a cross-sectional study were evaluated in the JBI checklist: sampling methods, study population, data collection, and analysis methods.^26^ The assessment was carried out by authors who had undergone extensive training by experienced epidemiologists and used a four-choice response scale (yes, no, unclear and not applicable). A score of one was assigned to an ‘yes’ response, while a score of zero was given for all other responses. A higher total score on the JBI checklist indicates a lower RoB.

### Certainty of evidence

We used the Grading of Recommendations, Assessment, Development and Evaluation (GRADE) method to determine the certainty of evidence.^27^ The GRADE method examines five domains: RoB, imprecision, inconsistency, indirectness and publication bias.^28^ By applying the rules of downgrading and upgrading within the GRADE method, we were able to rate the certainty levels of evidence for the pooled prevalence of PGD and its symptoms separately. The rating levels used were high, moderate, low or very low.

### Statistical analysis

Meta-analysis of prevalence data was conducted using the ‘metaprop’ package in R V.4.1.2. This analysis employed one-step generalised linear mixed models with the Logit link function, as recommended by Lin and Chu.^29^ Heterogeneity among studies was evaluated using the I^2^ statistic. When I^2^ was less than 50%, a fixed-effects model was used to obtain combined estimates. If I^2^ was 50% or greater, a random-effects model was employed. Subgroup analysis was carried out to identify potential sources of heterogeneity in the prevalence estimates. The Q-value test was employed to assess the significance of differences in estimated PRs across various subgroups. These subgroups were defined based on the study site, type of bereavement, average age, proportion of men in the study sample, survey method and the assessment of PGD or its symptoms. Publication bias was evaluated using funnel plots and Egger’s test. A two-sided p value of less than 0.05 was considered statistically significant.

As some of the included studies provided and directly compared subgroup-specific data—for instance, comparing PRs of PGD between males and females, and comparing time since loss (expressed as mean and SD) between grieving and non-grieving bereaved individuals—we performed a meta-analysis of these comparative data. This allowed us to better characterise individuals with PGD or its symptoms. For example, if the meta-analysis identified significantly higher PGD prevalence in females than in males, it could indicate that bereaved women are more likely to experience PGD than bereaved men. We conducted meta-analyses of both dichotomous and continuous outcomes using the ‘metabin’ and ‘metacont’ packages in R software V.4.1.2. Prevalence ratios and standardised mean differences (SMDs) were used as corresponding effect size measures.^30^

## Results

### Characteristics of the included studies

**Figure 1:**
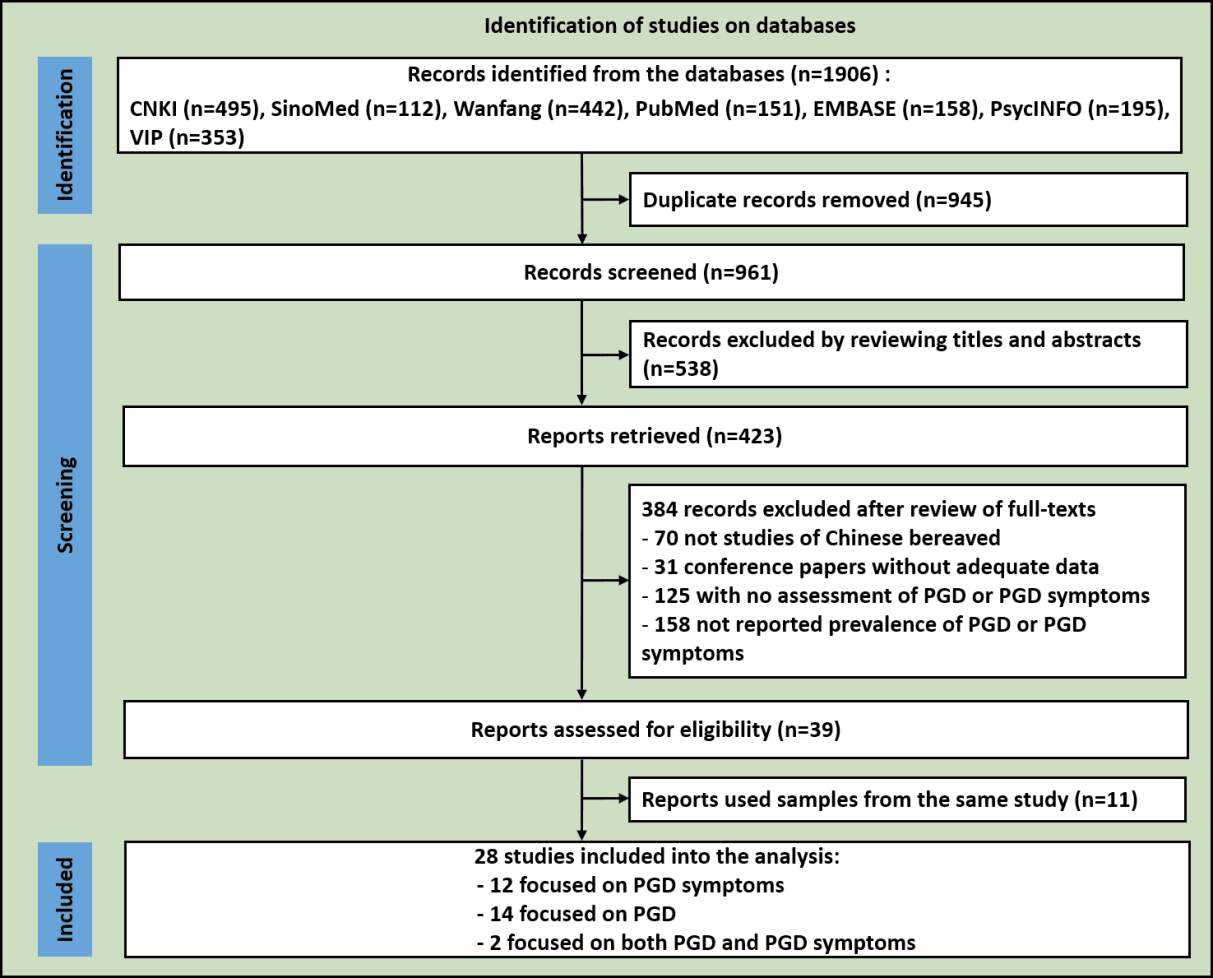
Flowchart of the study inclusion for the meta-analysis of the prevalence of PGD and its symptoms among bereaved Chinese. PGD, prolonged grief disorder.

The flowchart of study inclusion is shown in figure 1. Twenty-eight studies involving 10 994 bereaved persons were included: 14 focused solely on PGD,^8 17 19 20 31–40^ 12 focused solely on PGD symptoms,^15 16 41–50^ and two on both PGD and its symptoms.^18 51^ Detailed characteristics of the included studies are displayed in online supplemental material appendix 3.

### RoB of the included studies

The JBI checklist scores of the included studies ranged from 3 to 7, with a median score of 6. The two most common methodological problems among the included studies were a problematic sample frame (n=23) and an inappropriate sampling method (n=23).

### Meta-analysis of the prevalence of PGD and its symptoms

**Figure 2:**
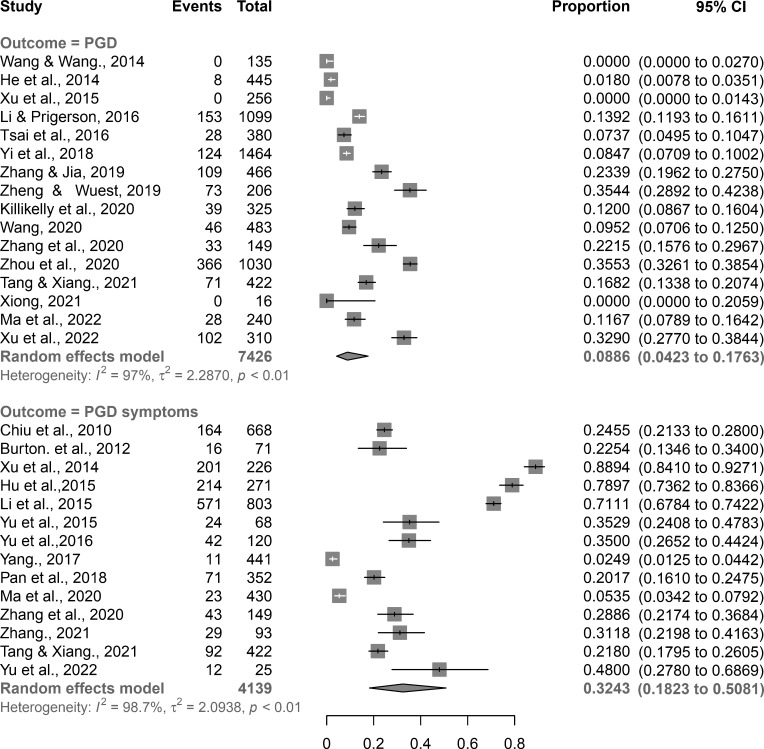
Forest plot of meta-analyses of PGD and its symptoms among bereaved Chinese. CI, confidence interval; PGD, prolonged grief disorder.

The combined PRs of PGD and its symptoms were 8.9% (95% confidence interval (CI): 4.2% to 17.6%) and 32.4% (95% CI: 18.2% to 50.8%), respectively (figure 2).

In comparison to men, women exhibited significantly higher PRs of both PGD (12.6% vs 8.4%, PR: 1.84, p<0.001) and its symptoms (29.6% vs 20.1%, PR: 1.69, p<0.001) (table 1). Additionally, women had higher PGD symptom scores compared with men (SMD: 0.24, p=0.031). Individuals diagnosed with PGD had a significantly shorter duration since the loss compared with those without PGD (SMD: −0.39, p<0.001). Moreover, individuals with religious beliefs had statistically higher PGD symptom scores than those without (SMD: 0.25, p=0.016), while people who lost loved ones due to violent causes scored higher on PGD symptom scales compared with those whose deceased loved ones died due to illnesses (SMD: 0.45, p=0.010) (online supplemental appendix 4).

**Table 1:** Meta-analyses of the prevalence rates of prolonged grief disorder and its symptoms by subpopulations of the bereaved Chinese and their corresponding prevalence ratios

Subpopulation		Number of studies	Sample size	Number of grieving individuals	Pooled prevalence (95% CI), %	PR (95% CI), %	P
PGD							
Sex	Female	5	1799	228	12.62 (4.89 to 28.71)*	1.84 (1.19 to 2.85)	<0.001
	Male		1042	85	8.41 (4.32 to 15.71)*	1	
Religious beliefs	Yes	5	482	64	18.54 (9.68 to 32.52)*	1.58 (0.96 to 2.60)*	0.071
	No		2367	249	9.90 (3.63 to 24.09)*	1	
Marital status	Married	4	666	100	13.57 (5.54 to 29.71)*	1.03 (0.79 to 1.34)	0.832
	Others		716	89	9.11 (2.60 to 27.93)*	1	
Kinship to the deceased	Child	2	287	43	15.01 (11.32 to 19.61)*	6.72 (0.30 to 151.13)*	0.230
	Spouse		213	25	11.70 (8.10 to 16.81)	3.90 (0.29 to 59.77)*	0.306
	Parent		458	32	3.63 (0.71 to 16.84)*	1	
Cause of death of the deceased	Violent	4	375	68	15.72 (8.20 to 28.03)*	1.17 (0.70 to 1.96)*	0.550
	Illness		973	121	10.61 (2.77 to 32.64)*	1	
Sex of the deceased	Male	3	602	116	20.11 (10.51 to 35.10)*	1.01 (0.78 to 1.31)	0.974
	Female		336	65	18.93 (10.12 to 32.73)*	1	
PGD symptoms							
Sex	Female	3	500	148	29.61 (25.80 to 33.81)	1.69 (1.29 to 2.23)	<0.001
	Male		307	56	20.14 (12.34 to 31.21)*	1	

*Random effects models.

CI, confidence interval; PGD, prolonged grief disorder; PR, prevalence ratios.

### Publication bias among the included studies

As depicted in online supplemental material appendix 5, the funnel plot for the meta-analysis of PGD was visually asymmetrical, while the funnel plot for the meta-analysis of PGD symptoms was visually symmetrical. Nonetheless, the results of Egger’s test indicated no statistically significant publication bias across the 16 studies regarding PGD (z=1.50, p=0.067) or the 14 studies regarding PGD symptoms (z=0.50, p=0.308).

### Sources of heterogeneity in the meta-analysis of the prevalence of PGD and its symptoms

As displayed in table 2 and table 3, study site, type of bereavement and diagnostic criteria for PGD were significant sources of heterogeneity in the meta-analysis of the prevalence of PGD, while study site, type of bereavement and mean age of the study sample were significant sources of heterogeneity in the meta-analysis of the prevalence of PGD symptoms. Specifically, surveys conducted solely online (18.1%) and offline (15.2%) had a significantly higher PGD prevalence than those conducted both online and offline (1.0%). Among bereaved participants, those who had deceased loved ones as their only children had the highest prevalence of PGD (22.7%), followed by those who experienced loss due to COVID-19 (16.8%) and earthquakes (8.5%). Bereaved individuals who experienced loss due to illnesses had the lowest prevalence of PGD (1.7%). In addition, surveys using the ICD-11 diagnostic criteria for PGD yielded a significantly higher PGD prevalence than those using the Prigerson *et al’s* 52 criteria (22.9% vs 5.8%, p=0.010).

**Table 2:** Subgroup analysis of the source of heterogeneity of the meta-analysis of prevalence of prolonged grief disorder

Subpopulation by variable		Number of studies	Sample size	Number of grieving individuals	Heterogeneity, I^2^ (%), P	Pooled prevalence (95% CI), %	Q (P)
Study site	Online and offline	3	836	8	0.03, 1.00	0.97 (0.51 to 1.92)	
	Online	4	2052	336	94.90, <0.001	18.10 (11.62 to 27.16)	4.04 (0.031)
	Offline	9	4538	836	97.82, <0.001	15.23 (9.63 to 23.33)	4.26 (0.039)
Type of the bereavement*	Death due to illness	7	2656	228	86.01, <0.001	1.74 (0.32 to 10.44)	
	Death due to earthquake	1	1464	124	Not applicable	8.52 (7.21 to 10.00)	2.96 (0.085)
	Death of the only child	7	2884	757	95.91, <0.001	22.68 (15.63 to 31.84)	8.02 (0.005)
	Death due to COVID-19	1	422	71	Not applicable	16.83 (13.58 to 20.72)	6.30 (0.012)
Sample size†	≤325	8	1637	275	90.14, <0.001	4.21 (0.64 to 25.87)	
	>325	8	5789	905	98.31, <0.001	11.69 (6.52 to 20.10)	0.98 (0.322)
Sampling method	Convenience	13	6554	1073	97.32, <0.001	7.40 (2.73 to 18.61)	
	Probability	3	872	107	87.57, <0.001	13.41 (8.72 to 20.00)	1.26 (0.263)
% of males among the study sample†	≤41.3	8	4309	773	98.32, <0.001	14.83 (7.53 to 27.31)	
	>41.3	8	3117	407	82.43, <0.001	3.65 (0.64 to 18.02)	2.43 (0.119)
Mean age of the study sample‡	18–35 years	4	1208	118	92.61, <0.001	5.88 (1.81 to 17.52)	
	36–59 years	4	1941	254	96.01, <0.001	5.43 (0.54 to 38.46)	0.01 (0.941)
	60+years	8	4277	808	97.84, <0.001	13.34 (6.11 to 26.90)	1.38 (0.240)
Assessment of PGD	PG-13	12	5270	815	97.38, <0.001	6.17 (2.14 to 17.03)	
	Others	4	2156	365	95.42, <0.001	17.80 (11.81 to 26.04)	3.58 (0.058)
Survey method	Self-report	12	5417	995	96.56, <0.001	8.93 (3.42 to 21.37)	
	Interview	4	2009	185	89.70, <0.001	9.83 (5.31 to 17.52)	0.03 (0.863)
Diagnostic criteria	Prigerson *et al* 52	12	5339	602	94.73, <0.001	5.81 (2.13 to 14.96)	
	ICD-11	4	2087	578	96.84, <0.001	22.89 (14.34 to 34.55)	6.67 (0.010)
JBI checklist score	3–5	6	1534	254	95.12, <0.001	5.01 (0.72 to 28.64)	
	6–7	10	5892	926	96.70, <0.001	10.90 (5.61 to 20.03)	0.58 (0.446)

*Studies that included participants bereaved by the loss of loved ones due to a variety of causes of death, primarily illnesses, were categorised as ‘death due to illness’. Studies that included participants bereaved by the loss of loved ones solely due to earthquakes, AIDS and COVID-19 were categorised as ‘death due to earthquake’, ‘death due to AIDS’ and ‘death due to COVID-19’, respectively. Studies that included participants who lost their only child, regardless of the causes of death, were categorised as ‘death of the only child’.

†The two continuous variables were dichotomised at the median value.

‡Studies with the mean age of the sample being ‘18–35 years’, ‘36–59 years’ and ‘60+years’ were crudely categorised into studies with predominantly young, middle-aged and older adults, respectively.

CI, confidence interval; ICD-11, International Classification of Diseases and Related Health Problems, 11th Edition; JBI checklist, Joanna Briggs Institute Critical Appraisal Checklist for Studies Reporting Prevalence Data; PG-13, Prolonged Grief Disorder-13; PGD, prolonged grief disorder.

**Table 3:** Subgroup analysis of the source of heterogeneity of the meta-analysis of prevalence of prolonged grief disorder symptoms

Subpopulation by variable		Number of studies	Sample size	Number of grieving individuals	Heterogeneity, I^2^ (%), P	Pooled prevalence (95% CI), %	Q (P)
Study site	Online and offline	2	871	34	77.76, 0.030	3.83 (2.22 to 6.31)	
	Online	4	870	191	68.34, 0.020	22.00 (19.32 to 24.80)	45.68 (<0.001)
	Offline	8	2398	1288	98.61, <0.001	50.92 (31.91 to 65.60)	44.28 (<0.001)
Type of the bereavement*	Death due to illness	6	2055	314	95.80, <0.001	13.60 (6.42 to 26.71)	
	Death due to earthquake	3	1300	986	93.42, <0.001	80.38 (70.27 to 87.71)	44.83 (<0.001)
	Death due to AIDS	2	188	66	0.01, 0.970	35.11 (28.63 to 42.20)	7.43 (0.006)
	Death of the only child	1	149	43	Not applicable	28.90 (22.21 to 36.62)	4.17 (0.041)
	Death due to COVID-19	2	447	104	87.93, <0.001	30.72 (16.11 to 50.50)	2.93 (0.087)
Sample size†	>226	7	3387	1146	99.34, <0.001	24.00 (8.32 to 52.32)	
	≤226	7	752	367	96.22, <0.001	28.90 (25.11 to 61.64)	1.26 (0.262)
Sampling method	Convenience	12	3764	1269	98.72, <0.001	27.84 (15.31 to 45.02)	
	Probability	2	375	244	99.13, <0.001	64.42 (18.31 to 93.64)	1.87 (0.172)
% of males among the study sample†	≤37.0	7	2348	905	99.10, <0.001	28.91 (9.12 to 62.40)	
	>37.0	7	1791	608	97.43, <0.001	36.01 (23.42 to 50.94)	0.17 (0.680)
Mean age of the study sample‡	18–35 years	3	1293	126	97.61, <0.001	6.92 (2.28 to 19.01)	
	36–59 years	9	2345	1273	98.42, <0.001	49.74 (32.11 to 67.42)	13.81 (<0.001)
	60+years	2	501	114	77.48, <0.001	23.50 (18.12 to 30.01)	5.43 (0.020)
Assessment of PGD symptoms	ICG	11	3497	1362	98.90, <0.001	34.92 (17.00 to 58.51)	
	Others	3	642	151	34.86, 0.220	23.52 (20.41 to 27.02)	1.21 (0.272)
Survey method	Self-report	6	1682	381	98.81, <0.001	22.46 (7.01 to 52.74)	
	Interview	8	2457	1132	98.72, <0.001	41.10 (24.00 to 60.72)	1.21 (0.272)
JBI checklist score	3–5	7	1229	238	93.14, <0.001	24.92 (15.23 to 38.00)	
	6–7	7	2910	1275	99.20, <0.001	40.42 (14.91 to 72.33)	0.89 (0.347)

*Studies that included participants bereaved by the loss of loved ones due to a variety of causes of death, primarily illnesses, were categorised as ‘death due to illness’. Studies that included participants bereaved by the loss of loved ones solely due to earthquakes, AIDS and COVID-19 were categorised as ‘death due to earthquake’, ‘death due to AIDS’ and ‘death due to COVID-19’, respectively. Studies that included participants who lost their only child, regardless of the causes of death, were categorised as ‘death of the only child’.

†The two continuous variables were dichotomised at the median value.

‡Studies with the mean age of the sample being ‘18–35 years’, ‘36–59 years’ and ‘60+years’ were crudely categorised into studies with predominantly young, middle-aged and older adults, respectively.

CI, confidence interval; ICG, Inventory of Complicated Grief; PGD, prolonged grief disorder.

Similarly, surveys conducted solely online (22.0%) and offline (50.9%) had a significantly higher prevalence of PGD symptoms than those conducted both online and offline (3.8%). The highest prevalence of PGD symptoms was observed among bereaved participants whose loved ones died due to earthquakes (80.4%), followed by those who experienced loss due to AIDS (35.1%) and COVID-19 (30.7%). Additionally, individuals whose deceased loved ones were the only children (28.9%) and those whose loved ones died of illnesses (13.6%) also exhibited notable rates of PGD symptoms. Studies predominantly involving middle-aged participants (49.7%) and older adults (23.5%) demonstrated significantly higher PRs of PGD symptoms compared with studies predominantly involving young adult participants (6.9%).

### Certainty of the evidence for the pooled prevalence of PGD and its symptoms

All our included studies were cross-sectional studies, so we initially classified the certainty levels for both the pooled prevalence of PGD and its symptoms as ‘low’. However, given the considerable RoB, wide 95% CIs, high levels of heterogeneity and clinical diversity among the samples from the included studies in this meta-analysis, we ultimately rated the certainty levels for both outcomes as ‘very low’.

## Discussion

### Main findings

Characterising the prevalence of PGD and its symptoms in the context of Chinese culture has important nosological, clinical and management implications. The present study systematically reviewed and quantitatively analysed the PRs of PGD and its symptoms among bereaved individuals in China. First, we found a prevalence of 8.9% for PGD and a prevalence of 32.4% for its symptoms. The rates of both PGD and its symptoms were statistically higher in women compared with men. Second, we found that individuals whose deceased loved ones died from illnesses had the lowest prevalence of PGD and its symptoms. On the other hand, individuals whose deceased loved ones were the only child had the highest prevalence of PGD, and those whose deceased loved ones died due to earthquakes had the highest prevalence of PGD symptoms. Third, the study observed that individuals with PGD had a shorter duration since the loss compared with those without PGD. Finally, several study-level factors were found to be associated with the prevalence of PGD or its symptoms including the survey site, the diagnostic criteria used to define PGD and the mean age of the study sample.

The 8.9% prevalence of PGD suggests that a significant proportion of bereaved individuals in China meet the diagnostic criteria for PGD; additionally, the 32.4% prevalence of its symptoms indicates that a larger portion of this population experiences symptoms of complex grief, even though they may not meet the full diagnostic criteria for PGD. The higher prevalence of PGD and its symptoms, as well as the greater severity of PGD symptoms in women compared with men among the bereaved Chinese, is consistent with findings from previous international studies.^53 54^ This difference may be attributed to females’ vulnerability, their involvement in caregiving and nurturing roles and their tendency to express emotions more openly.^21 55 56^

The literature indicates that the risk of PGD or its symptoms is higher when the death of a loved one occurs unexpectedly, suddenly, violently or under traumatic circumstances.^57–61^ Previous meta-analyses of international studies have reported PRs of PGD following non-violent bereavement, loss of family members due to cancer, unnatural deaths and loss during the COVID-19 pandemic at 9.8%, 14.2%, 49.0% and 46.4%, respectively.^61–64^ The PRs of complicated grief among bereaved family members of patients with cancer in Japan and earthquake survivors in Iran were 14% and 76%, respectively.^65 66^ Similarly, the present study found that the nature of bereavement is a significant determinant of the prevalence of PGD and its symptoms among the bereaved Chinese population.

In general, deaths due to illnesses are often anticipated and not violent, which may result in lower PRs of PGD and its symptoms as well as lower levels of PGD symptoms in Chinese bereaved individuals. However, the sudden and violent loss of lives and significant damage to physical assets during natural disasters like earthquakes intensify feelings of grief in bereaved individuals. This heightened intensity can persist for an extended period of time, leading to a higher risk of PGD and its symptoms among those who have experienced earthquakes.

Due to the stigma, discrimination and misconceptions associated with HIV/AIDS, the loss associated with AIDS can lead to additional complications in the grief process.^46 67^ This may explain the high prevalence of PGD symptoms, as high as 35.1%, observed among Chinese individuals bereaved by AIDS-related loss in this study. Additionally, due to the one-child policy in China, the loss of the only child can be particularly devastating for the bereaved parents. These parents may experience self-blame, guilt and a sense of not fulfilling their filial duties to their ancestors due to their inability to continue the family line.^13^ Moreover, since adult children traditionally serve as the primary home carers for their elderly parents in China, losing the only child also means losing the major source of financial and emotional support.^21^ These sociocultural factors could contribute to the increased risk of PGD and its symptoms in individuals who experience the loss of their only child.

Our study found that PGD and its symptoms were highly prevalent among those who have lost loved ones to COVID-19, with rates as high as 16.8% and 30.7%, respectively. These findings highlight the critical need for postpandemic grief counselling services in China, as patients COVID-19 often passed away in isolated settings without the opportunity for loved ones to say goodbye. This is supported by previous findings that a lack of meaningful communication before or during death can increase the risk of PGD among bereaved family members.^68^

Consistent with the universal phenomenon that grief alleviates over time,^61^ we found that individuals with PGD were more likely to have experienced a more recent loss. The positive association between the severity of PGD symptoms and religious beliefs among bereaved Chinese aligns with the higher prevalence of mental health challenges observed in Chinese individuals with religious beliefs, compared with those without.^69 70^ This could be due to the fact that, in China, the world’s most atheistic country, people often turn to religions for assistance when facing mental health challenges such as complicated grief.^21^

Compared to the Prigerson *et al*’s criteria,^52^ the ICD-11 diagnostic criteria are more lenient,^36^ which might explain the lower prevalence of PGD when conducting a meta-analysis of studies using the Prigerson *et al*’s criteria compared with those using the ICD-11 criteria. The findings that the study site and mean age of the study sample were associated with the pooled prevalence of PGD and its symptoms highlight the importance of considering an appropriate sample frame when examining the epidemiology of PGD.

### Limitations

We acknowledge that this study has certain limitations. First, none of the included studies was rated as having a low RoB. The dynamic nature of the population of bereaved individuals and the challenges of reaching them through household-based surveys led to the majority of the included studies being conducted solely online or through a combination of online and offline approaches. This makes it challenging to ascertain the representativeness of the online samples, warranting caution when generalising the findings to the broader population of bereaved individuals in China. Second, due to the limited number of included studies focusing on bereavement due to COVID-19, AIDS and earthquakes, the ranking of PRs for PGD and its symptoms by type of bereavement in this study may not be robust enough. Third, the identification of high-risk subgroups for PGD and its symptoms in this study was based solely on the results of direct comparisons between subgroups, without adjusting for potential confounders. As a result, the findings regarding high-risk subgroups are preliminary and require further validation through additional studies.

### Implications

In China, mental health service resources remain insufficient to meet the growing needs of the general population.^71 72^ This inadequacy constitutes a significant barrier to accessing grief counselling and other mental health services.^73 74^ Specifically, grief counselling services in China are still in their early developmental stages, which results in an insufficient and uneven distribution of these services in hospitals and communities. Concerns have also been raised regarding the quality of grief counselling services offered within China’s medical institutions.^21^ Additionally, the challenges of providing culturally adapted services further complicate the task of addressing the needs of the bereaved. Notably, no established community-based system for screening, managing or referring PGD currently exists in China.^75^

Considering the sheer size of the bereaved population in China, along with an 8.9% prevalence of PGD and a 32.4% prevalence of PGD symptoms, the demand for grief counselling services could be exceptionally high. Our study emphasises the urgency of strengthening these services for the bereaved population in China. Strategies could include integrating services into primary care, developing a two-way referral system between mental health institutions and clinics and providing training for community mental health workers.

The shorter duration of grief observed in bereaved individuals with PGD implies that the early provision of grief counselling services could be more effective in preventing PGD. Services could include psychosocial support, regular PGD screening and facilitating psychiatric referrals when necessary. If these services targeted high-risk groups such as women, individuals with religious beliefs, those who lost their only child and those grieving severe losses, they could be more cost-effective.

Finally, the very low level of evidence certainty suggests that further large-scale, representative studies employing stringent methodology are necessary. These would yield more accurate prevalence data on PGD and its symptoms in the bereaved Chinese population.

## Data Availability

All data relevant to the study are included in the article.

## References

[1] Department of economic and social affairs population division, United Nations . Data portal, custom data acquired via website. United Nations: New York. Available: https://population.un.org/DataPortal/ [Accessed 31 May 2023].

[2] Cerel J , Brown MM , Maple M , et al . How many people are exposed to suicide? Not six. Suicide Life Threat Behav 2019;49:529–34. 10.1111/sltb.12450 29512876

[3] Verdery AM , Smith-Greenaway E , Margolis R , et al . Tracking the reach of COVID-19 kin loss with a bereavement multiplier applied to the United States. Proc Natl Acad Sci U S A 2020;117:17695–701. 10.1073/pnas.2007476117 32651279 PMC7395491

[4] Stroebe M , Schut H , Stroebe W . Health outcomes of bereavement. Lancet 2007;370:1960–73. 10.1016/S0140-6736(07)61816-9 18068517

[5] Wen F-H , Prigerson HG , Chou W-C , et al . How symptoms of prolonged grief disorder, posttraumatic stress disorder, and depression relate to each other for grieving ICU families during the first two years of bereavement. Crit Care 2022;26:336. 10.1186/s13054-022-04216-5 36320037 PMC9628049

[6] Eisma MC . Prolonged grief disorder in ICD-11 and DSM-5-TR: challenges and controversies. Aust N Z J Psychiatry 2023;57:944–51. 10.1177/00048674231154206 36748103 PMC10291380

[7] Xiong WT , Wu HM , Chen J . Research progress on the diagnostic evaluation and treatment of prolonged grief disorder. Neural Injury and Functional Reconstruction 2023;18:26. 10.16780/j.cnki.sjssgncj.20210852

[8] Killikelly C , Zhou N , Merzhvynska M , et al . Development of the international prolonged grief disorder scale for the ICD-11: measurement of core symptoms and culture items adapted for Chinese and German-speaking samples. J Affect Disord 2020;277:568–76. 10.1016/j.jad.2020.08.057 32896722

[9] Nwokoroku SC , Neil B , Dlamini C , et al . A systematic review of the role of culture in the mental health service utilisation among ethnic minority groups in the United Kingdom. Glob Ment Health (Camb) 2022;9:84–93. 10.1017/gmh.2022.2 36618728 PMC9806997

[10] Zhong B-L , Chiu HF-K . Ageism, dementia, and culture. Int Psychogeriatr 2023;35:1–2. 10.1017/S1041610223000029 36748652

[11] Ho SW , Brotherson SE . Cultural influences on parental bereavement in Chinese families. Omega (Westport) 2007;55:1–25. 10.2190/4293-202L-5475-2161 17877079

[12] Bai N , Yin M . Analysis on the death culture differences between China and the West from medical perspective. Medicine & Philosophy 2014;35:21–3. Available: https://www.semanticscholar.org/paper/Analysis-on-the-Death-Culture-Differences-between-Nin/c9f388afdcc78e2cd352b796e1691cd399a05d2f#citing-papers

[13] He L , Tang XF , Zhu ZY , et al . Great pain: qualitative research on grief reactions of parents who lost their single child. Chin J Clin Psychol 2014;22:792–8. 10.16128/j.cnki.1005-3611.2014.05.053

[14] Hsu M-T , Kahn DL , Yee D-H , et al . Recovery through reconnection: a cultural design for family bereavement in Taiwan. Death Stud 2004;28:761–86. 10.1080/07481180490483391 15446285

[15] Xu Y , Herrman H , Bentley R , et al . Effect of having a subsequent child on the mental health of women who lost a child in the 2008 Sichuan earthquake: a cross-sectional study. Bull World Health Organ 2014;92:348–55. 10.2471/BLT.13.124677 24839324 PMC4007123

[16] Yang K . Research on the psychological reactions and treatments of the bereaved. Southwest Medical University; 2017.

[17] Wang HY , Wang JH . A survey of the grief response among bereaved cancer patients. Chin J Cancer 2014;6:298–301.

[18] Tang S , Xiang Z . Who suffered most after deaths due to COVID-19? Prevalence and correlates of prolonged grief disorder in COVID-19 related bereaved adults. Global Health 2021;17:19. 10.1186/s12992-021-00669-5 33573673 PMC7877329

[19] Yi X , Gao J , Wu C , et al . Prevalence and risk factors of prolonged grief disorder among bereaved survivors seven years after the Wenchuan earthquake in China: a cross-sectional study. Int J Nurs Sci 2018;5:157–61. 10.1016/j.ijnss.2018.04.001 31406818 PMC6626251

[20] Wang W . The prevalence, comorbidity and risks of prolonged grief disorders among Chinese Shidu parents. Chinese Medical University; 2020.

[21] Yuan M-D , Wang Z-Q , Fei L , et al . Prevalence of prolonged grief disorder and its symptoms in Chinese parents who lost their only child: a systematic review and meta-analysis. Front Public Health 2022;10. 10.3389/fpubh.2022.1016160 PMC9550932 36238241

[22] Pan H , Liu F . The prevalence of complicated grief among Chinese people at high risk: a systematic review and meta-analysis. Death Stud 2021;45:480–90. 10.1080/07481187.2019.1648342 31402787

[23] Luo W , Zhong B-L , Chiu HF-K . Prevalence of depressive symptoms among Chinese university students amid the COVID-19 pandemic: a systematic review and meta-analysis. Epidemiol Psychiatr Sci 2021;30:e31. 10.1017/S2045796021000202 33766163 PMC8047400

[24] Zhang QQ , Li L , Zhong BL . Prevalence of insomnia symptoms in older Chinese adults during the COVID-19 pandemic: a meta-analysis. Front Med (Lausanne) 2021;8. 10.3389/fmed.2021.779914 PMC8634335 34869501

[25] Munn Z , Moola S , Riitano D , et al . The development of a critical appraisal tool for use in systematic reviews addressing questions of prevalence. Int J Health Policy Manag 2014;3:123–8. 10.15171/ijhpm.2014.71 25197676 PMC4154549

[26] Xie Q , Liu X-B , Xu Y-M , et al . Understanding the psychiatric symptoms of COVID-19: a meta-analysis of studies assessing psychiatric symptoms in Chinese patients with and survivors of COVID-19 and SARS by using the Symptom Checklist-90-Revised. Transl Psychiatry 2021;11:290. 10.1038/s41398-021-01416-5 34001863 PMC8127471

[27] Balshem H , Helfand M , Schünemann HJ , et al . GRADE guidelines: 3. Rating the quality of evidence. J Clin Epidemiol 2011;64:401–6. 10.1016/j.jclinepi.2010.07.015 21208779

[28] Guyatt GH , Oxman AD , Vist GE , et al . GRADE: an emerging consensus on rating quality of evidence and strength of recommendations. BMJ 2008;336:924–6. 10.1136/bmj.39489.470347.AD 18436948 PMC2335261

[29] Lin L , Chu H . Meta-analysis of proportions using generalized linear mixed models. Epidemiology 2020;31:713–7. 10.1097/EDE.0000000000001232 32657954 PMC7398826

[30] Tamhane AR , Westfall AO , Burkholder GA , et al . Prevalence odds ratio versus prevalence ratio: choice comes with consequences. Stat Med 2016;35:5730–5. 10.1002/sim.7059 27460748 PMC5135596

[31] Zheng Y , Wuest LG . Assessing the impact of factors on parental grief among older Chinese parents. Death Stud 2021;45:110–8. 10.1080/07481187.2019.1616854 31122149

[32] Li J , Prigerson HG . Assessment and associated features of prolonged grief disorder among Chinese bereaved individuals. Compr Psychiatry 2016;66:9–16. 10.1016/j.comppsych.2015.12.001 26995230

[33] Tsai W-I , Prigerson HG , Li C-Y , et al . Longitudinal changes and predictors of prolonged grief for bereaved family caregivers over the first 2 years after the terminally ill cancer patient’s death. Palliat Med 2016;30:495–503. 10.1177/0269216315603261 26311571

[34] Xu X , Wen J , Skritskaya NA , et al . Grief-related beliefs in Shidu parents with and without prolonged grief disorder: psychometric properties of a Chinese version of the typical beliefs questionnaire. Clin Psychol Psychother 2022;29:512–23. 10.1002/cpp.2641 34235799

[35] Ma H , Zhao S , Long M , et al . The relationship between culture-related grief beliefs, prolonged grief disorder and suicide ideation among Shidu parents in rural China. Clin Psychol Psychother 2023;30:54–63. 10.1002/cpp.2768 35776076

[36] Zhou N , Wen J , Stelzer E-M , et al . Prevalence and associated factors of prolonged grief disorder in Chinese parents bereaved by losing their only child. Psychiatry Res 2020;284:112766. 10.1016/j.psychres.2020.112766 31951871

[37] He L , Tang S , Yu W , et al . The prevalence, comorbidity and risks of prolonged grief disorder among bereaved Chinese adults. Psychiatry Res 2014;219:347–52. 10.1016/j.psychres.2014.05.022 24924526

[38] Zhang YD , Jia XM . Mental health status of the Shiduers: based on latent profile analysis. J Clin Psychol 2019;27:362–6. 10.16128/j.cnki.1005-3611.2019.02.031

[39] Xiong BX . Reconstruction of meaning after the death of grandparents in skip-generation raising group: a qualitative study based on adult attachment interview. Huazhong Normal University; 2021.

[40] Xu W , He L , Fu ZF , et al . The prolonged grief disorder symptoms and their predictive factors among bereaved individuals. J Clin Psychol 2015;23:277–80. 10.16128/j.cnki.1005-3611.2015.02.019

[41] Chiu Y-W , Huang C-T , Yin S-M , et al . Determinants of complicated grief in caregivers who cared for terminal cancer patients. Support Care Cancer 2010;18:1321–7. 10.1007/s00520-009-0756-6 19816716

[42] Burton CL , Yan OH , Pat-Horenczyk R , et al . Coping flexibility and complicated grief: a comparison of American and Chinese samples. Depress Anxiety 2012;29:16–22. 10.1002/da.20888 21898713 PMC3242921

[43] Hu X-L , Li X-L , Dou X-M , et al . Factors related to complicated grief among bereaved individuals after the Wenchuan earthquake in China. Chin Med J (Engl) 2015;128:1438–43. 10.4103/0366-6999.157647 26021497 PMC4733770

[44] Li J , Chow AYM , Shi Z , et al . Prevalence and risk factors of complicated grief among Sichuan earthquake survivors. J Affect Disord 2015;175:218–23. 10.1016/j.jad.2015.01.003 25645702

[45] Yu NX , Chan CLW , Zhang J , et al . Resilience and vulnerability: prolonged grief in the bereaved spouses of marital partners who died of AIDS. AIDS Care 2016;28:441–4. 10.1080/09540121.2015.1112354 26573556

[46] Xiaonan Yu N , Chow AYM , Chan CLW , et al . Stigma never dies: mourning a spouse who died of AIDS in China. Psychiatry Res 2015;230:968–70. 10.1016/j.psychres.2015.10.034 26553145

[47] Yu Z , Liang J , Guo L , et al . Psychosocial intervention on the dual-process model for a group of COVID-19 bereaved individuals in Wuhan: a pilot study. Omega (Westport) 2022. 10.1177/00302228221083067 PMC8958304 35341382

[48] Ma X , Li Q , Zeng C , et al . Influence of grief cognition on prolonged grief response: the moderator role of whether students were medical students or not. Chin J Health Psychol 2020;28:1532–7. 10.13342/j.cnki.cjhp.2020.10.022

[49] Zhang MS . Analysis of influencing factors about grief response and stress disorder of family members of patients dead in hospital. Shandong University; 2021.

[50] Pan H , Cheung CK , Hu J . Intimacy and complicated grief among Chinese elders having lost their spouses: mediating role of meaning making. J Loss Trauma 2018;23:244–58. 10.1080/15325024.2018.1435367

[51] Zhang H , Shang Z , Wu L , et al . Prolonged grief disorder in Chinese Shidu parents who have lost their only child. Eur J Psychotraumatol 2020;11. 10.1080/20008198.2020.1726071 PMC7048219 32158517

[52] Prigerson HG , Horowitz MJ , Jacobs SC , et al . Prolonged grief disorder: psychometric validation of criteria proposed for DSM-V and ICD-11. PLoS Med 2009;6:e1000121. 10.1371/journal.pmed.1000121 19652695 PMC2711304

[53] Lundorff M , Bonanno GA , Johannsen M , et al . Are there gender differences in prolonged grief trajectories? A registry-sampled cohort study. J Psychiatr Res 2020;129:168–75. 10.1016/j.jpsychires.2020.06.030 32739617

[54] Maccallum F , Lundorff M , Johannsen M , et al . An exploration of gender and prolonged grief symptoms using network analysis. Psychol Med 2023;53:1770–7. 10.1017/S0033291721003391 34503594

[55] Shulla RM , Toomey RB . Sex differences in behavioral and psychological expression of grief during adolescence: a meta-analysis. J Adolesc 2018;65:219–27. 10.1016/j.adolescence.2018.04.001 29674176

[56] Chen WC , Chen SJ , Zhong BL . Sense of alienation and its associations with depressive symptoms and poor sleep quality in older adults who experienced the lockdown in Wuhan, China, during the COVID-19 pandemic. J Geriatr Psychiatry Neurol 2022;35:215–22. 10.1177/08919887221078564 35130783 PMC8899829

[57] Szuhany KL , Malgaroli M , Miron CD , et al . Prolonged grief disorder: course, diagnosis, assessment, and treatment. Focus (Am Psychiatr Publ) 2021;19:161–72. 10.1176/appi.focus.20200052 34690579 PMC8475918

[58] Milman E , Neimeyer RA , Fitzpatrick M , et al . Prolonged grief symptomatology following violent loss: the mediating role of meaning. Eur J Psychotraumatol 2017;8. 10.1080/20008198.2018.1503522 PMC6095024 30128081

[59] Doering BK , Barke A , Vogel A , et al . Predictors of prolonged grief disorder in a German representative population sample: unexpectedness of bereavement contributes to grief severity and prolonged grief disorder. Front Psychiatry 2022;13. 10.3389/fpsyt.2022.853698 PMC9090313 35558417

[60] Glickman K . Prolonged grief disorder in a diverse college student sample. Front Psychol 2020;11. 10.3389/fpsyg.2020.604573 PMC7830516 33505337

[61] Djelantik A , Smid GE , Mroz A , et al . The prevalence of prolonged grief disorder in bereaved individuals following unnatural losses: systematic review and meta regression analysis. J Affect Disord 2020;265:146–56. 10.1016/j.jad.2020.01.034 32090736

[62] Lundorff M , Holmgren H , Zachariae R , et al . Prevalence of prolonged grief disorder in adult bereavement: a systematic review and meta-analysis. J Affect Disord 2017;212:138–49. 10.1016/j.jad.2017.01.030 28167398

[63] Kustanti CY , Jen H-J , Chu H , et al . Prevalence of grief symptoms and disorders in the time of COVID-19 pandemic: a meta-analysis. Int J Ment Health Nurs 2023;32:904–16. 10.1111/inm.13136 36880520

[64] Kustanti CY , Chu H , Kang XL , et al . Prevalence of grief disorders in bereaved families of cancer patients: a meta-analysis. Palliat Med 2022;36:305–18. 10.1177/02692163211066747 34965780

[65] Aoyama M , Sakaguchi Y , Morita T , et al . Factors associated with possible complicated grief and major depressive disorders. Psychooncology 2018;27:915–21. 10.1002/pon.4610 29247587

[66] Ghaffari-Nejad A , Ahmadi-Mousavi M , Gandomkar M , et al . The prevalence of complicated grief among BAM earthquake survivors in Iran. Arch Iran Med 2007;10:525–8. 17903061

[67] Sikkema KJ , Kochman A , DiFranceisco W , et al . AIDS-related grief and coping with loss among HIV-positive men and women. J Behav Med 2003;26:165–81. 10.1023/a:1023086723137 12776385

[68] Otani H , Yoshida S , Morita T , et al . Meaningful communication before death, but not present at the time of death itself, is associated with better outcomes on measures of depression and complicated grief among bereaved family members of cancer patients. J Pain Symptom Manage 2017;54:273–9. 10.1016/j.jpainsymman.2017.07.010 28711756

[69] Huang JX , Xu YM , Zhong BL . Relationship between Buddhist belief and suicide risk in Chinese persons undergoing methadone maintenance therapy for heroin dependence. Front Psychiatry 2020;11:414. 10.3389/fpsyt.2020.00414 32457671 PMC7221180

[70] Liang Y-J , Deng F , Liang P , et al . Suicidal ideation and mental health help-seeking behaviors among older Chinese adults during the COVID-19 pandemic. J Geriatr Psychiatry Neurol 2022;35:245–51. 10.1177/08919887221078568 35139677 PMC8844439

[71] Zhang H-G , Fan F , Zhong B-L , et al . Relationship between left-behind status and cognitive function in older Chinese adults: a prospective 3-year cohort study. Gen Psychiatr 2023;36:e101054. 10.1136/gpsych-2023-101054 37337546 PMC10277132

[72] Zhong B-L , Zhou D-Y , He M-F , et al . Mental health problems, needs, and service use among people living within and outside Wuhan during the COVID-19 epidemic in China. Ann Transl Med 2020;8:1392. 10.21037/atm-20-4145 33313137 PMC7723535

[73] Zhong BL , Xu YM , Li Y . Prevalence and unmet need for mental Healthcare of major depressive disorder in community-dwelling Chinese people living with vision disability. Front Public Health 2022;10. 10.3389/fpubh.2022.900425 PMC9257003 35812506

[74] Zhong BL , Xiang YT . Challenges to and recent research on the mental health of older adults in China during the COVID-19 pandemic. J Geriatr Psychiatry Neurol 2022;35:179–81. 10.1177/08919887221078558 35245997 PMC8899836

[75] Yuan LX , Zhou Y , Tang QB , et al . Development of grief counselling in Hong Kong and its implications for Mainland China. Medicine and Philosophy 2016;37:31–3. 10.12014/j.issn.1002-0772.2016.02a.08

