# Supplementary material for: Prevalence of prolonged grief disorder and its symptoms among bereaved individuals in China: a systematic review and meta-analysis
Source: Gen Psychiatr. 2024 Mar 5;37(2):e101216. doi: 10.1136/gpsych-2023-101216 (PMC10916091; doi:10.1136/gpsych-2023-101216)
Supplement: Supplementary data [file gpsych-2023-101216supp001.pdf]

BMJ Publishing Group Limited (BMJ) disclaims all liability and responsibility arising from any reliance placed on this supplemental material which has been supplied by the author(s)

Prevalence of prolonged grief disorder and its symptoms among bereaved individuals in China: a systematic review and meta-analysis

Supplementary materials

Appendix 1. PRISMA checklist

Appendix 2. Literature search strategies

Appendix 3. Characteristics of included studies

Appendix 4. Standardised mean differences in age and age of the deceased between bereaved Chinese individuals with and without PGD or its symptoms, as well as in PGD symptom scores between subpopulations based on sociodemographic characteristics

Appendix 5. Funnel plot of publication bias

Yuan M-D, *et al. General Psychiatry* 2024; 37:e101216. doi: 10.1136/gpsych-2023-101216

Appendix 1: PRISMA checklist

Section and Topic	Item #	Checklist item	Location where item is reported
**TITLE**			
Title	1	Identify the report as a systematic review.	Title page
**ABSTRACT**			
Abstract	2	See the PRISMA 2020 for Abstracts checklist.	Abstract p3
**INTRODUCTION**			
Rationale	3	Describe the rationale for the review in the context of existing knowledge.	pp5-6
Objectives	4	Provide an explicit statement of the objective(s) or question(s) the review addresses.	p6
**METHODS**			
Eligibility criteria	5	Specify the inclusion and exclusion criteria for the review and how studies were grouped for the syntheses.	p7
Information sources	6	Specify all databases, registers, websites, organisations, reference lists and other sources searched or consulted to identify studies. Specify the date when each source was last searched or consulted.	p7
Search strategy	7	Present the full search strategies for all databases, registers and websites, including any filters and limits used.	Appendix 2
Selection process	8	Specify the methods used to decide whether a study met the inclusion criteria of the review, including how many reviewers screened each record and each report retrieved, whether they worked independently, and if applicable, details of automation tools used in the process.	p7
Data collection process	9	Specify the methods used to collect data from reports, including how many reviewers collected data from each report, whether they worked independently, any processes for obtaining or confirming data from study investigators, and if applicable, details of automation tools used in the process.	p8
Data items	10a	List and define all outcomes for which data were sought. Specify whether all results that were compatible with each outcome domain in each study were sought (eg for all measures, time points, analyses), and if not, the methods used to decide which results to collect.	p8
	10b	List and define all other variables for which data were sought (eg participant and intervention characteristics, funding sources). Describe any assumptions made about any missing or unclear information.	p8
Study risk of bias assessment	11	Specify the methods used to assess risk of bias in the included studies, including details of the tool(s) used, how many reviewers assessed each study and whether they worked independently, and if applicable, details of automation tools used in the process.	p8
Effect measures	12	Specify for each outcome the effect measure(s) (eg risk ratio, mean difference) used in the synthesis or presentation of results.	pp8-9
Synthesis methods	13a	Describe the processes used to decide which studies were eligible for each synthesis (eg tabulating the study intervention characteristics and comparing against the planned groups for each synthesis (item #5)).	p9

Section and Topic	Item #	Checklist item	Location where item is reported
	13b	Describe any methods required to prepare the data for presentation or synthesis, such as handling of missing summary statistics, or data conversions.	p9
	13c	Describe any methods used to tabulate or visually display results of individual studies and syntheses.	p9
	13d	Describe any methods used to synthesize results and provide a rationale for the choice(s). If meta-analysis was performed, describe the model(s), method(s) to identify the presence and extent of statistical heterogeneity, and software package(s) used.	p9
	13e	Describe any methods used to explore possible causes of heterogeneity among study results (eg subgroup analysis, meta-regression).	p9
	13f	Describe any sensitivity analyses conducted to assess robustness of the synthesized results.	Not applicable
Reporting bias assessment	14	Describe any methods used to assess risk of bias due to missing results in a synthesis (arising from reporting biases).	p9
Certainty assessment	15	Describe any methods used to assess certainty (or confidence) in the body of evidence for an outcome.	p9
**RESULTS**			
Study selection	16a	Describe the results of the search and selection process, from the number of records identified in the search to the number of studies included in the review, ideally using a flow diagram.	Figure1
	16b	Cite studies that might appear to meet the inclusion criteria, but which were excluded, and explain why they were excluded.	Figure1
Study characteristics	17	Cite each included study and present its characteristics.	Appendix 3
Risk of bias in studies	18	Present assessments of risk of bias for each included study.	Appendix 3
Results of individual studies	19	For all outcomes, present, for each study: (a) summary statistics for each group (where appropriate) and (b) an effect estimate and its precision (eg confidence/credible interval), ideally using structured tables or plots.	Figures 2,
Results of syntheses	20a	For each synthesis, briefly summarise the characteristics and risk of bias among contributing studies.	Table 1
	20b	Present results of all statistical syntheses conducted. If meta-analysis was done, present for each the summary estimate and its precision (eg confidence/credible interval) and measures of statistical heterogeneity. If comparing groups, describe the direction of the effect.	pp10-11
	20c	Present results of all investigations of possible causes of heterogeneity among study results.	pp10-11<br>Table 2-3
	20d	Present results of all sensitivity analyses conducted to assess the robustness of the synthesized results.	Not applicable
Reporting biases	21	Present assessments of risk of bias due to missing results (arising from reporting biases) for each synthesis assessed.	p10,

Section and Topic	Item #	Checklist item	Location where item is reported
			Appendix 5
Certainty of evidence	22	Present assessments of certainty (or confidence) in the body of evidence for each outcome assessed.	p11
**DISCUSSION**			
Discussion	23a	Provide a general interpretation of the results in the context of other evidence.	pp11-12
	23b	Discuss any limitations of the evidence included in the review.	p14
	23c	Discuss any limitations of the review processes used.	p14
	23d	Discuss implications of the results for practice, policy, and future research.	pp14-15
**OTHER INFORMATION**			
Registration and protocol	24a	Provide registration information for the review, including register name and registration number, or state that the review was not registered.	p6
	24b	Indicate where the review protocol can be accessed, or state that a protocol was not prepared.	p6
	24c	Describe and explain any amendments to information provided at registration or in the protocol.	Not applicable
Support	25	Describe sources of financial or non-financial support for the review, and the role of the funders or sponsors in the review.	p2
Competing interests	26	Declare any competing interests of review authors.	p2
Availability of data, code and other materials	27	Report which of the following are publicly available and where they can be found: template data collection forms; data extracted from included studies; data used for all analyses; analytic code; any other materials used in the review.	Not applicable

Yuan M-D, *et al. General Psychiatry* 2024; 37:e101216. doi: 10.1136/gpsych-2023-101216

Appendix 2. Literature search strategies

Databases	Search strategies	Publication date
Chinese language		
China National Knowledge Infrastructure	（篇关摘："丧亲" + "丧子" + "丧偶" + "失独" + "去世" + "失去" + "自杀"（精确））AND（篇关摘："哀伤" + "持续性复杂丧亲障碍"（精确））	Inception to October 4, 2023
SinoMed	("丧亲"[摘要] OR "丧子"[摘要] OR "丧偶"[摘要] OR "失独"[摘要] OR "去世"[摘要] OR "失去"[摘要] OR "自杀"[摘要]) AND ("哀伤"[摘要] OR "持续性复杂丧亲障碍"[摘要])	Inception to October 4, 2023
Wanfang data	主题:("哀伤" or "持续性复杂丧亲障碍") and 主题:("丧亲" or "丧子" or "丧偶" or "失独" or "去世" or "失去" or "自杀")	Inception to October 4, 2023
VIP Information	R=("丧亲" + "丧子" + "丧偶" + "失独" + "去世" + "失去" + "自杀") and R=("持续性复杂丧亲障碍" + "哀伤")	Inception to October 4, 2023
English language		
PubMed	((grief*[Title/Abstract]) AND ((((((((bereave*[Title/Abstract]) OR (mourn*[Title/Abstract])) OR (sorrow[Title/Abstract])) OR (surviv*[Title/Abstract])) OR (Shidu[Title/Abstract])) OR (los*[Title/Abstract])) OR (death[Title/Abstract])) OR (die*[Title/Abstract])) OR (suicide*[Title/Abstract]))) AND ((((Taiwan[Title/Abstract]) OR (Chin*[Title/Abstract])) OR (Hong Kong[Title/Abstract])) OR (Macau[Title/Abstract]))	Inception to October 4, 2023
Embase	grief*:ab,ti AND (bereave*:ab,ti OR mourn*:ab,ti OR sorrow:ab,ti OR surviv*:ab,ti OR shidu:ab,ti OR los*:ab,ti OR death:ab,ti OR die*:ab,ti OR suicide*:ab,ti) AND (chin*:ab,ti OR taiwan:ab,ti OR 'hong kong':ab,ti OR macau:ab,ti)	Inception to October 4, 2023

PsycInfo	(AB Chin* OR AB Taiwan OR AB Hong Kong OR AB Macau) AND (AB bereave* OR AB mourn* OR AB sorrow OR AB surviv* OR AB Shidu OR AB los* OR AB death OR AB die* OR AB suicide*) AND (AB grief*)	Inception to October 4, 2023

Appendix 3. Characteristics of included studies

Study	Reference	Channel of participants recruitment	Survey period	Event causing the bereavement	Number of survey completers	Sampling method	Response rate (%)	Numbers of men and women	Age of the sample (years)	Instrument	Data collection	Diagnostic criteria of prolonged grief disorder (PGD)	Number of subjects with PGD (%)	JBI checklist score*
Burton. et al., 2012	[1]	Advertisements, referrals, and mailings in Hong Kong, China	NR	The death of spouses due to multiple causes	71	Convenience sampling	NR	9/62	Mean: 45.6±7.4	PG-11	In-person interview	Shear et al., 2011	S: 16 (22.5)	3
Chiu et al., 2010	[2]	A hospice in Taiwan, China	2007–2008	Death due to cancer	668	Convenience sampling	73.0	263/405	Mean: 42.9±10.5	ICG	Telephone interview	ICG>25	S: 164 (24.6)	6
He et al., 2014	[3]	Universities, hospitals, and communities and online websites in mainland China	March 2012–September 2013	Death due to multiple causes, mainly illnesses	445	Convenience sampling	50.0	86/349	Mean: 27.6±11.8	PG-13	Online self-report	Prigerson et al., 2009	D: 8 (1.8)	5
Hu et al.,2015	[4]	Communities in three hardest-hit areas in Sichuan, China	November 2009–February 2010	Death due to earthquake	271	Convenience sampling	94.4	123/148	<40: 108<br>40–49: 84<br>50–59: 29<br>≥60: 50	ICG	Paper-pencil self-report	ICG>25	S: 214 (79.0)	6

Yuan M-D, *et al. General Psychiatry* 2024; 37:e101216. doi: 10.1136/gpsych-2023-101216

Killikelly et al., 2020	[5]	Online websites in mainland China	NR	Death due to multiple causes, mainly illnesses	325	Convenience sampling	NR	104/212	Mean: 33.1±12.3	IPGDS	Online self-report	ICD-11	D: 39 (12.7)	6
Li et al., 2015	[6]	A temporary shelter in Beichuan, China	2009	Death due to earthquake	803	Convenience sampling	93.5	297/506	Mean: 46.7±15.5	ICG	In-person interview	ICG>25	S: 571(71.1)	6
Li & Prigerson, 2016	[7]	Online websites in mainland China	NR	Death due to multiple causes, mainly illnesses	1099 (1358)*	Convenience sampling	NR	665/693	Mean: 41.9±11.2	ICG	Online self-report	Prigerson et al., 2009	D: 153 (13.9)	6
Ma et al., 2020	[8]	Online websites and communities in Shandong and Yunnan, China	NR	Death due to multiple causes, mainly illnesses	430	Convenience sampling	NR	141/289	NR (participants were university students)	ICG-R	Self-report	ICG-R>102	S: 23 (5.4)	5
Ma et al., 2022	[9]	Communities in China	November 2019–February 2020	Death of the only child	240	Probability sampling	87.3	113/127	Mean: 62.9±6.2	PG-13	Paper-pencil self-report	Prigerson et al., 2009	D: 28 (11.7)	7

Pan et al., 2018	[10]	Communities in Yongjia, China	NR	Death of spouses due to multiple causes, mainly illnesses	352	Convenience sampling	NR	76/276	Mean: 77.6±8.7	ICG	In-person interview	ICG>25	S: 71(20.2)	6
Tang & Xiang., 2021	[11]	Online websites and mobile applications	September 1, 2020–October 3, 2020	Death due to COVID-19	422	Convenience sampling	88.7	234/188	Mean: 32.7±9.3	IPGDS	Online self-report	ICD-11 IPGDS>42.5	D: 71 (37.8) S: 92 (48.9)	4
Tsai et al., 2016	[12]	A medical center in Taiwan, China	January 2007–December 2012	Death due to cancer	380 (493)*	Convenience sampling	77.1	174/319	Mean: 50.8±18.0	PG-13	In-person interview	Prigerson et al., 2009	D: 28 (7.4)	6
Wang &Wang., 2014	[13]	Local communities and online websites in mainland China	NR	Death due to cancer	135	Convenience sampling	NR	74/61	NR	PG-13	Online and paper-pencil self-report	Prigerson et al., 2009	D: 0 (0.0)	3
Wang, 2020	[14]	Communities in Shenyang, mainland China	March 2017–September 2017	Death of the only child	483	Probability sampling	81.2	201/282	49-59: 185 60+: 298	PG-13	Paper-pencil self-report	Prigerson et al., 2009	D: 46 (9.5)	7

Yuan M-D, *et al. General Psychiatry* 2024; 37:e101216. doi: 10.1136/gpsych-2023-101216

Xiong, 2021	[15]	Communities in China	NR	Death due to multiple causes, mainly illnesses	16	Convenience sampling	NR	7/9	Mean: 23.8±2.8	PG-13	Online and in-person interview	Prigerson et al., 2009	D: 0 (0.0)	4
Xu et al., 2014	[16]	Communities in Dujiangyan, China	October 2010–March 2011	The death of a child due to earthquake	226	Probability sampling	81.4	0/226	Mean: 39.82	ICG	In-person interview	ICG>25	S: 201 (88.9)	6
Xu et al., 2015	[17]	Local communities and online websites in mainland China	NR	Death due to cancer	256	Convenience sampling	91.8	113/143	Mean: 39.2±18.9	PG-13	Paper-pencil and online self-report	Prigerson et al., 2009	D: 0 (0.0)	6
Xu et al., 2022	[18]	Public interest organization in China	December 2019–January 2020	Death of the only child	310	Convenience sampling	NR	92/218	Mean: 61.7±5.5	SCI-CG	Computer-assisted self-report	ICD-11	D: 102 (32.9)	4
Yang., 2017	[19]	Online websites, universities, communities, and hospitals in China	November 2015–June 2016	Death of family member due to multiple causes, mainly illnesses	441	Convenience sampling	88.2	113/328	<18: 69<br>18-30: 256<br>31-40: 33<br>41-50: 53<br>51+: 30	ICG-R	Online and paper-pencil self-report	ICG-R>102	S:11 (2.5)	6
Yi et al., 2018	[20]	Three towns in Wenchuan, mainland China	NR	Death due to earthquake	1464	Convenience sampling	97.6	604/860	≤40:345<br>41-60:542<br>≥61:577	PG-13	In-person or telephone interview	Prigerson et al., 2009	D: 124 (8.5)	6
Yu et al., 2015	[21]	Villages in Henan, China	June–October 2011	Death of spouses due to AIDS	68	Convenience sampling	82.9	35/33	Mean: 49.8±9.1	ICG	In-person interview	ICG≥30	S: 24(35.3)	5

Yu et al.,2016	[22]	Villages in Henan, China	June–October 2011	Death of spouses due to AIDS	120	Convenience sampling	NR	58/62	≤45: 46<br>46-55: 43<br>>55: 31	ICG	In-person interview	ICG≥30	S: 42(35.0)	5
Yu et al., 2022	[23]	Social media platforms in Wuhan, China	NR	Death due to COVID-19	25	Convenience sampling	59.5	5/20	Mean: 43.7 ±11.0	ICG	Online self-report	ICG>48	S: 12(48.0)	4
Zhang et al., 2020	[24]	Communities in Shanghai, China	September 2015–January 2017	Death of the only child	149	Probability sampling	96.1	59/90	Mean: 62.3±4.9	PG-13<br>PG-11	In-person interview	Prigerson et al., 2009<br>PG-11>36	D: 33 (22.2)<br>S: 43 (28.9)	7
Zhang., 2021	[25]	A general hospital in Shandong, China	July 1, 2019–June 30, 2020	Death due to multiple illnesses	93	Convenience sampling	100.0	71/22	Median: 48	ICG	Paper-pencil self-report	ICG>25	S: 29 (31.2)	4
Zhang & Jia, 2019	[26]	Communities and social work organizations in mainland China	June 2017–December 2017	Death of the only child	466	Convenience sampling	93.8	212/254	Mean: 60.2±7.0	PG-13	Paper-pencil self-report	Prigerson et al., 2009	D: 109 (23.4)	6
Zheng & Wuest, 2019	[27]	Online bereaved parents' support groups in China	NR	Death of the only child	206	Convenience sampling	NR	71/135	Mean: 56.9±6.3	PG-13	Online self-report	Prigerson et al., 2009	D: 73 (35.5)	4
Zhou et al., 2020	[28]	Communities, support groups, public interest organization, and government agencies in China	April 2017–May 2018	Death of the only child	1030	Convenience sampling	97.7	381/643	Mean: 59.9±7.3	PG-13 + SCL-90	Paper-pencil self-report	ICD-11	D: 366 (35.5)	7

Note: NR, not reported; PG-13, Prolonged Grief Disorder-13; ICG: Inventory of Complicated Grief; ICG-R: Revised version of Inventory of Complicated Grief; D: Clinical diagnosis of Prolonged Grief Disorder; S: Symptoms of Prolonged Grief Disorder; JBI checklist: the Joanna Briggs Institute Critical Appraisal Checklist for Studies Reporting Prevalence Data; IPGDS, International ICD-11 Prolonged Grief Disorder Scale; SCI-CG, the Structured Clinical Interview for Complicated Grief; SCL-90, the Symptom Checklist-90.

References:

1. Burton CL, Yan OH, Pat-Horenczyk R, et al. Coping flexibility and complicated grief: a comparison of American and Chinese samples. *Depress Anxiety* 2012;**29**:16-22.

2.Chiu YW, Huang CT, Yin SM, et al. Determinants of complicated grief in caregivers who cared for terminal cancer patients. *Support Care Cancer* 2010;**18**:1321-7.

3. He L, Tang S, Yu W, et al. The prevalence, comorbidity and risks of prolonged grief disorder among bereaved Chinese adults. *Psychiatry Res* 2014;**219**:347-52.

4. Hu XL, Li XL, Dou XM, et al. Factors Related to Complicated Grief among Bereaved Individuals after the Wenchuan Earthquake in China. *Chin Med J (Engl)* 2015;**128**:1438-43.

5. Killikelly C, Zhou N, Merzhvynska M, et al. Development of the international prolonged grief disorder scale for the ICD-11: Measurement of core symptoms and culture items adapted for chinese and german-speaking samples. *J Affect Disord* 2020;**277**:568-76.

6. Li J, Chow AY, Shi Z, et al. Prevalence and risk factors of complicated grief among Sichuan earthquake survivors. *J Affect Disord* 2015;**175**:218-23.

7. Li J, Prigerson HG. Assessment and associated features of prolonged grief disorder among Chinese bereaved individuals. *Compr Psychiatry* 2016;**66**:9-16.

8. Ma X, Li Q, Zeng C, et al. Influence of grief cognition on peolonged grief response: the moderator role of whether students were medical students or not. *China Journal of Health Psychology* 2020;**28**:1532-37.

9. Ma H, Zhao S, Long M, et al. The relationship between culture-related grief beliefs, prolonged grief disorder and suicide ideation among Shidu parents in rural China. *Clinical Psychology & Psychotherapy* 2022;**doi: 10.1002/cpp.2768**.

10. Pan H, Cheung CK, Hu J. Intimacy and Complicated Grief among Chinese Elders Having Lost their Spouses: Mediating Role of Meaning Making. *Journal of Loss and Trauma* 2018;**23**:244-58.

11. Tang S, Xiang Z. Who suffered most after deaths due to COVID-19? Prevalence and correlates of prolonged grief disorder in COVID-19 related bereaved adults. *Global Health* 2021;**17**:19.

12. Tsai WI, Prigerson HG, Li CY, et al. Longitudinal changes and predictors of prolonged grief for bereaved family caregivers over the first 2 years after the terminally ill cancer patient's death. *Palliat Med* 2016;**30**:495-503.

13. Wang HY, Wang JH. A survey of the grief response among bereaved cancer patients. *Chinese Journal of Oncology Prevention and Treatment* 2014;**6**:298-301.

14. Wang W. The prevalence, comorbidity and risks of prolonged grief disorders among Chinese shidu parents. Chinese Medical University, 2020.

15. Xiong BX. Reconstruction of meaning after the death of grandparents in skip-generation raising group: a qualitative study based on adult attachment interview. Huazhong Normal University, 2021.

16. Xu Y, Herrman H, Bentley R, et al. Effect of having a subsequent child on the mental health of women who lost a child in the 2008 Sichuan earthquake: a cross-sectional study. *Bull World Health Organ* 2014;**92**:348-55.

17. Xu W, He L, Fu ZF, et al. The prolonged grief disorder symptoms and their predictive factors among bereaved individuals. *Chinese Journal of Clinical Psychology* 2015;**23**:277-80.

18. Xu X, Wen J, Skritskaya NA, et al. Grief-related beliefs in shidu parents with and without prolonged grief disorder: Psychometric properties of a Chinese version of the Typical Beliefs Questionnaire. *Clin Psychol Psychother* 2022;**29**:512-23.

19. Yang K. Research on the Psychological Reactions and Treatments of the Bereaved. Southwest Medical University, 2017.

20. Yi X, Gao J, Wu C, et al. Prevalence and risk factors of prolonged grief disorder among bereaved survivors seven years after the Wenchuan earthquake in China: A cross-sectional study. *Int J Nurs Sci* 2018;**5**:157-61.

21. Xiaonan Yu N, Chow AY, Chan CL, et al. Stigma never dies: Mourning a spouse who died of AIDS in China. *Psychiatry Res* 2015;**230**:968-70.

22. Yu NX, Chan CL, Zhang J, et al. Resilience and vulnerability: prolonged grief in the bereaved spouses of marital partners who died of AIDS. *AIDS Care* 2016;**28**:441-4.

23. Yu Z, Liang J, Guo L, et al. Psychosocial Intervention on the Dual-Process Model for a Group of COVID-19 Bereaved Individuals in Wuhan: A Pilot Study. *Omega (Westport)* 2022:302228221083067.

24. Zhang H, Shang Z, Wu L, et al. Prolonged grief disorder in Chinese Shidu parents who have lost their only child. *Eur J Psychotraumatol* 2020;**11**:1726071.

25. Zhang MS. Analysis of influencing factors about grief response and stress disorder of family members of patients dead in hospital. Shandong University, 2021.

26.Zhang YD, Jia XM. Mental health status of the Shiduers: based on latent profile analysis. *Chinese Journal of Clinical Psychology* 2019;**27**:362-66.

27. Zheng Y, Wuest LG. Assessing the impact of factors on parental grief among older Chinese parents. *Death Stud* 2019;**45**:110-18.

28. Zhou N, Wen J, Stelzer EM, et al. Prevalence and associated factors of prolonged grief disorder in Chinese parents bereaved by losing their only child. *Psychiatry Res* 2020;**284**:112766.

Appendix 4. Standardised mean differences in age and age of the deceased between bereaved Chinese individuals with and without PGD or its symptoms, as well as in PGD symptom scores between subpopulations based on sociodemographic characteristics

Subpopulation		Number of studies	Sample size	SMD (95%CI)	P
Age					
PGD	Yes	3	143	0.21 (-0.66–1.08)*	0.588
	No	3	716		
PGD symptoms	Yes	3	204	0.12 (-0.42–0.67)*	0.655
	No	3	603		
Time since the loss					
PGD	Yes	3	143	-0.39 (-0.58–-0.19)	<0.001
	No	3	716		
Age of the deceased					
PGD	Yes	3	143	-0.20 (-1.10–0.70)*	0.627
	No	3	716		
PGD symptom score					
Sex	Female	6	1013	0.24 (0.02–0.46)*	0.031
	Male	6	705		
Marital status	Married	6	1110	-0.02 (-0.18–0.14)*	0.789
	Other	6	603		
Religious beliefs	Yes	3	129	0.25 (0.05–0.45)	0.016
	No	3	583		
Kinship to the deceased	Spouse	3	80	0.26 (-0.59–1.11)*	0.876
	Child	3	265		
Cause of death of the deceased	Violent	5	553	0.45 (0.14–0.77)*	0.010
	Illness	5	892		
Sex of the deceased	Men	3	628	-0.08 (-0.32–0.16)*	0.427
	Women	3	285		

*random-effects model

Note: CI, confidence interval; PGD, prolonged grief disorder; SMD, Standardised mean differences.

Yuan M-D, *et al. General Psychiatry* 2024; 37:e101216. doi: 10.1136/gpsych-2023-101216
